# Development and validation of nomogram models for predicting immune-related adverse events in recurrent and metastatic nasopharyngeal carcinoma patients treated with PD-L1 inhibitors

**DOI:** 10.3389/fonc.2025.1539514

**Published:** 2025-03-13

**Authors:** Mengyuan Liu, Zheran Liu, Shuangshuang He, Yiyan Pei, Shihong Xu, Junyou Ge, Yan Qing, Youneng Wei, Ye Chen, Ping Ai, Xingchen Peng

**Affiliations:** ^1^ Division of Head & Neck Tumor Multimodality Treatment, Cancer Center, West China Hospital, Sichuan University, Chengdu, China; ^2^ Department of Targeting Therapy & Immunology, Cancer Center, West China Hospital, Sichuan University, Chengdu, China; ^3^ Sichuan Kelun-Biotech Biopharmaceutical Co. Ltd., Chengdu, China; ^4^ Division of Abdominal Tumor Multimodality Treatment, Department of Radiation Oncology, Cancer Center, West China Hospital, Sichuan University, Chengdu, China

**Keywords:** NPC, irAEs, PD-L1 inhibitors, biomarkers, nomogram

## Abstract

**Objective:**

To predict the incidence of immune-related Adverse Events (irAEs) in patients with recurrent or metastatic Nasopharyngeal Carcinoma (NPC) treated with Programmed Death-Ligand 1 (PD-L1) inhibitors, this study developed and validated nomogram models incorporating demographic, clinical, and biological variables.

**Methods:**

Data from 153 NPC patients were analyzed, incorporating variables including age, sex, Body Mass Index (BMI), clinical stage, and biomarkers. Predictive models were constructed using multivariable logistic regression, Least Absolute Shrinkage and Selection Operator (LASSO) regression, and Ridge regression. The models’ performance was evaluated using Receiver Operating Characteristic (ROC) curves, calibration curves, and Decision Curve Analysis (DCA). Internal validation was conducted through k-fold cross-validation.

**Results:**

Independent predictors of irAEs included PD-L1, Free Thyroxine (FT4), Sodium (Na), and lymphocyte counts. Of the three models, the stepwise regression model performed best, with an area under the curve (AUC) of 0.78. Calibration curves showed a strong correlation between predicted and observed outcomes, and DCA demonstrated high clinical utility.

**Conclusion:**

The nomogram models effectively predict irAEs in NPC patients treated with PD-L1 inhibitors. Early identification of patients with elevated PD-L1, abnormal FT4, Na, or irregular lymphocyte counts allows for closer monitoring and personalized treatment, potentially improving outcomes. Further research is required to confirm these findings across other cancer types and therapies.

## Introduction

NPC is prevalent in southern China, Southeast Asia, and North Africa, with nonkeratinizing differentiated and undifferentiated carcinoma being the dominant pathological subtype and often associated with Epstein–Barr Virus (EBV) infection (1). At the time of diagnosis, most NPC patients are already in the advanced stages of the disease, with approximately 10% presenting with distant metastases. Recurrent or distant metastasis, either at initial diagnosis or following treatment, remains the primary cause of treatment failure in patients with NPC (2). Several clinical studies have confirmed the efficacy of combining immunotherapy with chemotherapy for the first-line treatment of recurrent and metastatic NPC, as well as the effectiveness of immunotherapy as a second-line or later treatment. However, most studies report response rates ranging between 20% and 30%, with some patients experiencing severe adverse reactions (3). These irAEs can range from mild to life-threatening and affect various organs and systems, including the skin, gastrointestinal tract, lungs, and endocrine glands. Failure to detect and manage severe irAEs can lead to long-term damage or even treatment discontinuation. This highlights the clinical importance of monitoring and identifying biomarkers associated with irAEs to mitigate potential harm.

Recent studies have highlighted peripheral blood biomarkers in assessing and predicting the efficacy of and adverse reactions to immunotherapy (4). Research across various cancers, including non-small cell lung cancer, metastatic renal cancer, osteosarcoma, melanoma, and head and neck squamous cell carcinoma, has demonstrated that specific changes in peripheral blood indices—namely the neutrophil-to-lymphocyte ratio (NLR), platelet-to-lymphocyte ratio (PLR), platelet-to-albumin ratio (PAR), lymphocyte-to-monocyte ratio (LMR), hemoglobin (HB), absolute lymphocyte count (ALC), absolute neutrophil count (ANC), and nutritional status—can significantly influence treatment efficacy and the occurrence of irAEs (2, 3, 5). However, research on biomarkers for predicting the efficacy and irAEs, especially in NPC, is limited. The POLARIS-02 study revealed that baseline plasma EBV DNA titers and their dynamic changes were significantly correlated with Progression-Free Survival (PFS), Overall Survival (OS), and Durable Clinical Benefit (DCB, defined as PFS ≥6 months) in patients with advanced NPC undergoing immunotherapy. We plan to develop a comprehensive predictive model specifically for NPC patients treated with PD-L1. This model incorporates some biomarkers to improve the prediction of irAEs, enabling clinicians to more effectively manage the risks associated with immunotherapy.

## Method

### Data source

This study used data from an open-label, multicenter phase 2 clinical trial conducted between 2017 and 2019 at 42 hospitals in China involving 153 patients with NPC (**Supplementary Figure 1**) (6). The inclusion criteria required patients to have recurrent or metastatic nonkeratinizing NPC, have failed at least two lines of chemotherapy, and meet the following conditions: being over 18 years old, having an Eastern Cooperative Oncology Group (ECOG) performance status of 0–1, and a life expectancy of at least 12 weeks. Patients with prior immunotherapy treatments, central nervous system metastasis, or active autoimmune diseases were excluded from the study. The trial followed ethical guidelines, and all participants provided written informed consent. Blood samples were taken at baseline stages to analyze biomarkers, and irAEs were monitored and graded according to established clinical guidelines throughout the study.

Patients in this study received KL-A167, a PD-L1 inhibitor, at a fixed dose of 900 mg via intravenous infusion every two weeks. Treatment was continued until one of the following occurred: confirmed disease progression, unacceptable toxicity, or withdrawal of informed consent. The decision to confirm Progressive Disease (PD) was based on repeat evaluations performed at least four weeks after the initial assessment, at the investigator’s discretion. For patients who discontinued treatment due to reasons other than documented disease progression, tumor assessments were conducted until PD, initiation of a new antitumor therapy, loss to follow-up, or death. No dose reductions of KL-A167 were permitted, and treatment was permanently discontinued if irAEs did not resolve to grade 0–1 within 12 weeks following the last dose. IrAEs were monitored from screening through the treatment period and up to 30 days after the withdrawal visit. This study primarily aimed to develop predictive models for identifying patients at risk of irAEs, thus enabling more effective early interventions and improving clinical outcomes.

### Variables

In this study, we collected demographic data, including age, sex, and Body Mass Index (BMI), along with clinical characteristics, namely clinical stage, liver metastasis status, smoking history, and alcohol consumption history. Baseline biological indicators encompassed blood and immune parameters, including Red Blood Cell (RBC), Hemoglobin (HGB), Platelet (PLT), White Blood Cell (WBC), NLR, PLR, and EBV DNA, as well as liver and kidney function, lipid levels, thyroid function, and other laboratory data. Continuous variables were converted into categorical variables on the basis of cutoff values determined via the "cutoff" package in R. We used the Akaike Information Criterion (AIC) to select the most influential variables, which were then included in the logistic regression analysis. The primary goal of the study was to assess the type and severity of immune-related adverse events (irAEs) that occur in NPC patients during follow-up.

The study incorporated a range of demographic, clinical, and biological variables, each selected for its clinical significance and potential association with immune-related adverse events (irAEs). All variables were initially treated as continuous; where necessary, cutoff values were derived using ROC curve analysis to enhance predictive accuracy. This methodological approach ensured that variable definitions were not only statistically robust but also clinically relevant.

### Construction of the nomogram

Three nomogram prediction models were constructed via different variable selection methods. Initially, univariate logistic regression analysis was performed, and variables with p values < 0.2 were included in the multivariate logistic regression analysis. For each variable, the version (continuous or categorical) with the smaller AIC value was selected (7). Multivariate logistic regression analysis identified Na (≤ 140.05", "> 140.05"), FT4, and PD_L1 as independent risk factors. The first prediction model was developed by selecting variables on the basis of univariate logistic regression analysis, where those with p values < 0.05 were included in the multivariate analysis. Variables with p values < 0.1 from the multivariate analysis were subsequently used to construct the model (8). For the second prediction model, we applied bidirectional stepwise regression to select the most significant variables. Finally, the third prediction model combined univariate logistic regression analysis with LASSO regression (9). Here, the LASSO method was used with a penalty factor (α) to filter out overlapping variables, which were then incorporated into the final model (10).

### Model performance and validation

The internal validation of this study was conducted via k-fold cross-validation, and the performance of the models was assessed through ROC curves, DCA curves, and calibration curves (11). The ROC curve was employed to evaluate the model's discriminative ability, with the area under the curve (AUC) reflecting the accuracy of the predictions. Calibration curves were used to assess the agreement between the predicted probabilities and the actual outcomes, ensuring the model's reliability. DCA was utilized to estimate the clinical "net benefit" of the predictive model in comparison with the default strategies of treating all patients or treating none, thereby providing valuable insights into the practical application of the model in clinical decision-making.

### Statistical analysis

Candidate predictors were identified through univariate analysis (P < 0.10), followed by LASSO regression for feature selection. Significant variables were further refined using multivariable logistic regression to construct the final nomogram model. Although our dataset contains fewer than 200 variables, the ratio of predictors to observations still poses a risk of overfitting. LASSO regression was applied to eliminate less informative variables by shrinking coefficients to zero, while Ridge regression was employed to reduce collinearity and improve model stability. The data analysis was performed via R software version 4.4.1. The R packages “dplyr (version 1.1.4)”, “survival” (version 3.3-1), “glm” (version 4.4.1), “rms” (version 6.8-1), “pROC” (version 1.18.5), and “ggplot2” (version 3.5.1) were used to develop and evaluate the model. The statistical significance of the two-sided p value was set at ≤ 0.05.

## Results

### Baseline characteristics

This study included 153 patients divided into two groups on the basis of the occurrence of irAEs: 105 patients did not experience irAEs, whereas 48 patients did. The baseline characteristics of these patients are summarized in **Table 1**. The two groups did not significantly differ in terms of age, BMI, ECOG performance status, sex, smoking and alcohol history, tumor stage, lymph node stage, or liver metastasis. Overall, the baseline characteristics of the patients in this study were predominantly male patients, with higher ECOG performance scores, no liver metastasis, and all patients receiving standard treatment protocols, including chemotherapy and/or radiotherapy. The differences in the distributions of all the baseline characteristics between the two groups were not statistically significant (all p > 0.05).

**Table 1:** Baseline characteristics of patients with and without immune-related adverse events (irAEs).

Variable	Overall (N=153)	Without irAEs (N=105)	With irAEs (N=48)	p value
Age (Mean [SD])	47.56 (9.81)	48.03 (10.12)	46.54 (9.12)	0.386
Body Mass Index (kg/m^2^)				
<18.5	29 (19.0%)	17 (16.2%)	12 (25.0%)	0.260
18.5–23.9	92 (60.1%)	63 (60.0%)	29 (60.4%)	
>23.9	32 (20.9%)	25 (23.8%)	7 (14.6%)	
ECOG_PS				
0	59 (38.6%)	42 (40.0%)	17 (35.4%)	0.718
1	94 (61.4%)	63 (60.0%)	31 (64.6%)	
Gender				
Male	125 (81.7%)	86 (81.9%)	39 (81.2%)	1.000
Female	28 (18.3%)	19 (18.1%)	9 (18.8%)	
Tumor Stage				
T0–T2	52 (34.0%)	42 (40.0%)	10 (20.8%)	0.059
T3–T4	50 (32.7%)	30 (28.6%)	20 (41.7%)	
Tx	51 (33.3%)	33 (31.4%)	18 (37.5%)	
Node Stage				
N0–N2	84 (54.9%)	55 (52.4%)	29 (60.4%)	0.537
N3	26 (17.0%)	20 (19.0%)	6 (12.5%)	
Nx	43 (28.1%)	30 (28.6%)	13 (27.1%)	
Liver Metastasis				
No	82 (53.6%)	54 (51.4%)	28 (58.3%)	0.535
Yes	71 (46.4%)	51 (48.6%)	20 (41.7%)	

### Occurrence of irAEs

In this study, 153 patients were analyzed, 48 (31.3%) of whom experienced irAEs (**Table 2**). The most common irAEs were endocrine-related and occurred in 20.9% (32 patients), followed by digestive system-related (7.8%, 12 patients) and cardiac-related events (6.5%, 10 patients). The majority of irAEs were mild, with 27.4% (42 cases) being grade 1–2. However, 3.9% (6 patients) were Grade 3 or higher, indicating more severe reactions. Endocrine irAEs were exclusively grade 1–2, whereas some cardiac (2 cases), digestive (3 cases), and hematologic (2 cases) irAEs progressed to grade 3 or higher. Other less frequent irAEs included dermatologic events, fatigue, and renal and metabolic disorders, most of which were mild (1.3% to 3.9%). These findings underscore the prevalence of mild irAEs but highlight the potential for more serious events, particularly in the cardiac, digestive, and hematologic systems, warranting close clinical attention (**Supplementary Figure 2**).

**Table 2:** Incidence and severity of immune-related adverse events (irAEs) among patients.

irAEs	Summary	Grade1_2	Grade3_4
Any irAEs	48 (31.3%)	42 (27.4%)	6 (3.9%)
Endocine irAEs	32 (20.9%)	32 (20.9%)	0
Cardiac irAEs	10 (6.5%)	8 (5.2%)	2 (1.3%)
Digestive System irAEs	12 (7.8%)	9 (5.9%)	3 (1.9%)
Hematology irAEs	6 (3.9%)	4 (2.6%)	2 (1.3%)
Dermatological irAEs	5 (3.2%)	5 (3.2%)	0
Fatigue	4 (2.6%)	4 (2.6%)	0
Renal irAEs	2 (1.3%)	2 (1.3%)	0
Metabolism irAEs	6 (3.9%)	5 (3.2%)	1 (0.7%)

### Univariate and multivariate logistic regression analyses

In this study, we first conducted univariate logistic regression analysis on each numerical variable. ROC curves were used to determine the optimal cutoff values, allowing us to convert these numerical variables into categorical variables. We then compared the AIC values of the continuous and categorical versions of the same variable, selecting the one with the lower AIC for further logistic regression analysis. Variables with a p value < 0.2 in the univariate analysis were subsequently included in the multivariate logistic regression analysis. The final results of both the univariate and multivariate logistic regression analyses are presented in the above table. In the multivariate analysis, variables with a p value < 0.05 were considered independent risk factors associated with the occurrence of irAEs during PD-L1 treatment. Specifically, the odds ratio (OR) for PD-L1 was 0.432 (95% CI: 0.188–0.993, p = 0.048), indicating that PD-L1 may play a significant independent role in the development of irAEs. Additionally, the dichotomized variables for FT4, Na, and lymphocyte count were also significant in the multivariate analysis. The OR for FT4 was 0.921 (95% CI: 0.851–0.996, p = 0.040), that for Na was 2.461 (95% CI: 1.109–5.459, p = 0.027), and that for the lymphocyte count was 2.768 (95% CI: 1.052–7.285, p = 0.039). These findings suggest that, in addition to PD-L1, FT4, Na, and lymphocyte count may also be potential factors influencing the occurrence of irAEs (**Table 3**). In summary, this study systematically selected and analyzed variables through regression analysis, identifying PD-L1 and several other important biomarkers as independent factors for irAEs.

**Table 3:** Univariate and multivariate logistic regression analyses of factors associated with immune-related adverse events (irAEs).

Variable	Univariable		Multivariable	
	OR (95% CI)	*P* value	OR (95% CI)	*P* value
Lymphocyte ≥1.32 VS < 1.32^*^	2.768 (1.052 – 7.285)	0.039		
FT4	0.936 (0.876 – 0.999)	0.049	0.921 (0.851 – 0.996)	0.040
Na ≥140.05 VS < 140.05^*^	1.997 (0.998 – 3.997)	0.051	2.461 (1.109 – 5.459)	0.027
LDL	1.323 (0.958 – 1.826)	0.089	1.405 (0.954 – 2.067)	0.085
PD _L1	0.555 (0.269 – 1.142)	0.110	0.432 (0.188 – 0.993)	0.048
NK Cells	0.974 (0.941 – 1.009)	0.140	0.966 (0.930 – 1.004)	0.080
NLR	0.933 (0.848 – 1.028)	0.161		
K	0.535 (0.213 – 1.344)	0.184		
APTT ≥29.45 VS <29.45^*^	1.586 (0.798 – 3.157)	0.189		
PT	1.227 (0.902 – 1.670)	0.192		

FT4, Free Thyroxine; LDL, Low Density Lipoprotein; PD_L1, Programmed Death-Ligand 1; NK Cells, Natural Killer Cells; NLR, Neutrophil-to-Lymphocyte Ratio; APTT, Activated Partial Thromboplastin Time; PT, Prothrombin Time.

*Utilize the area under the ROC curve to find the optimal cutoff value, incorporating variables with p values < 0.20 from the univariate logistic regression analysis into the multivariate logistic regression analysis.

### Construction of predictive models

**Figure 1:**
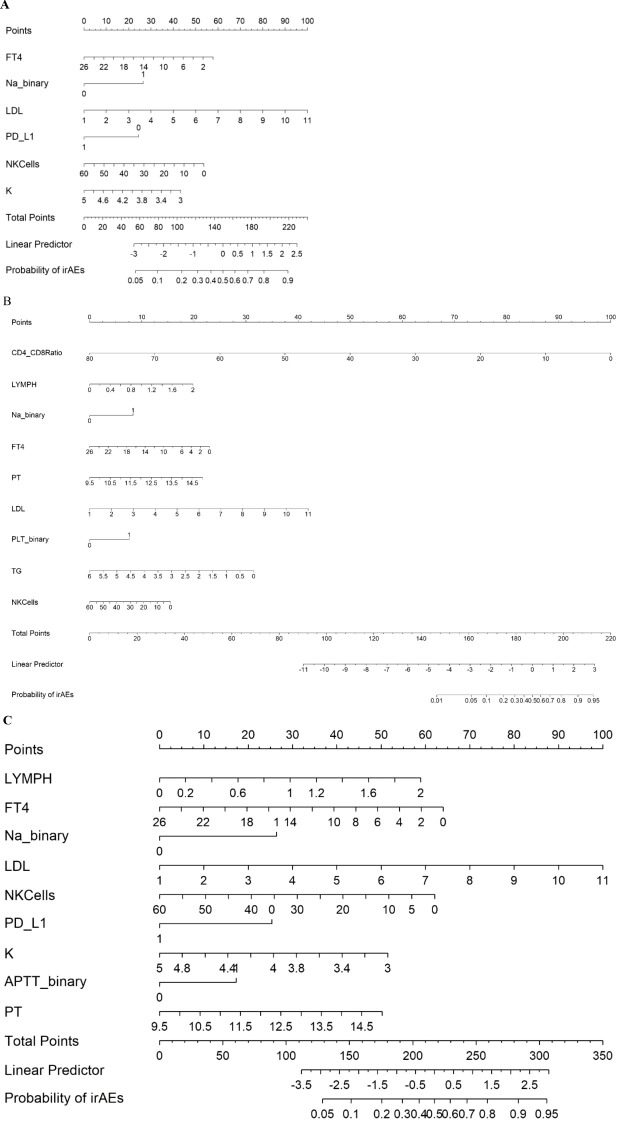
Nomogram predicting irAEs using Model 1 **(A)**, Model 2 **(B)**, and Model 3 **(C)**, FT4 (Free Thyroxine): pmol/L. Na_binary (Sodium): Binary variable with a cutoff of 140 mmol/L. LDL (Low-Density Lipoprotein): mmol/L. PD_L1 (Programmed Death-Ligand 1): Binary variable (0 = negative, 1 = positive). NKCells (Natural Killer Cells): cells/µL. K (Potassium): mmol/L. CD4_CD8 Ratio (CD4/CD8 Ratio). LYMPH (Lymphocytes): ×10^9^/L. PT (Prothrombin Time): seconds. PLT_binary (Platelets): Binary variable with a cutoff of 208 ×10^9^/L.TG (Triglycerides): mmol/L. APTT_binary (Activated Partial Thromboplastin Time): Binary variable with a cutoff of 29.4 seconds.

**Figure 2:**
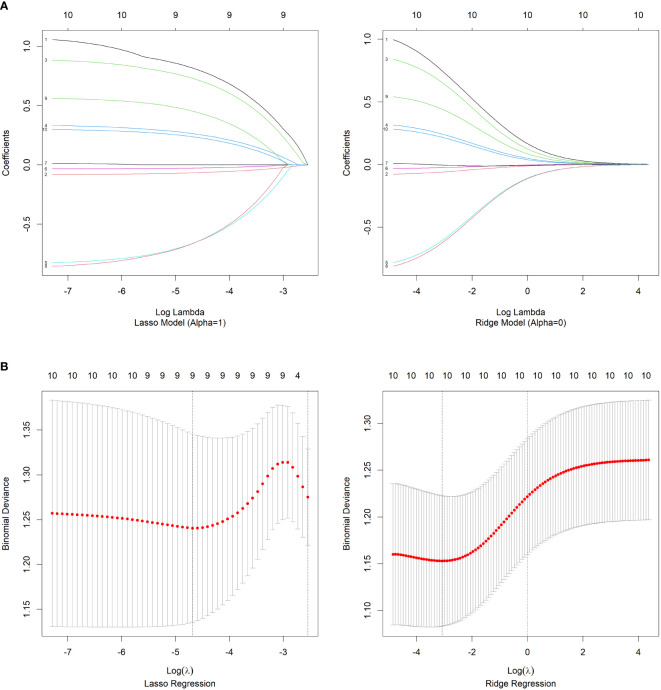
Regularization Path **(A)** and Cross-Validation **(B)** for LASSO and Ridge Regression Models.

In this study, we employed different methods to select variables and construct three logistic regression models to predict irAEs. First, we developed the first nomogram model by selecting variables from the multivariate logistic regression analysis with p values less than 0.1. These variables included FT4, Na binary, Low-Density Lipoprotein (LDL), PD_L1, Natural Killer Cells (NK cells), and Potassium (K) (**Figure 1A**). For the second model, we utilized a stepwise regression approach. We constructed a comprehensive model containing various potential influencing factors and applied forward, backward, and bidirectional stepwise regression methods to filter the variables. The selected variables for this model included the Cluster of Differentiation 4/8 Ratio (CD4/CD8), lymphocyte count, Na binary, FT4, Prothrombin Time (PT), LDL, PLT binary, Triglycerides (TG), and NK cells (**Figure 1B**). The third model was built via least absolute shrinkage and selection operator (LASSO) and ridge regression techniques (**Figure 2A**). These methods introduce regularization terms to reduce model complexity and mitigate the risk of overfitting. LASSO regression, through L1 regularization, enabled variable selection, whereas ridge regression, through L2 regularization, smoothed the variable coefficients (**Figure 2B**). Cross-validation was employed to determine the optimal lambda value, ultimately leading to the selection of key predictive variables, including lymphocyte count, FT4, Na-binary, LDL, NK cells, PD-L1, NLR, K, and PT (**Figure 1C**). These variables were then used to construct the final nomogram.

### Model performance validation

**Figure 3:**
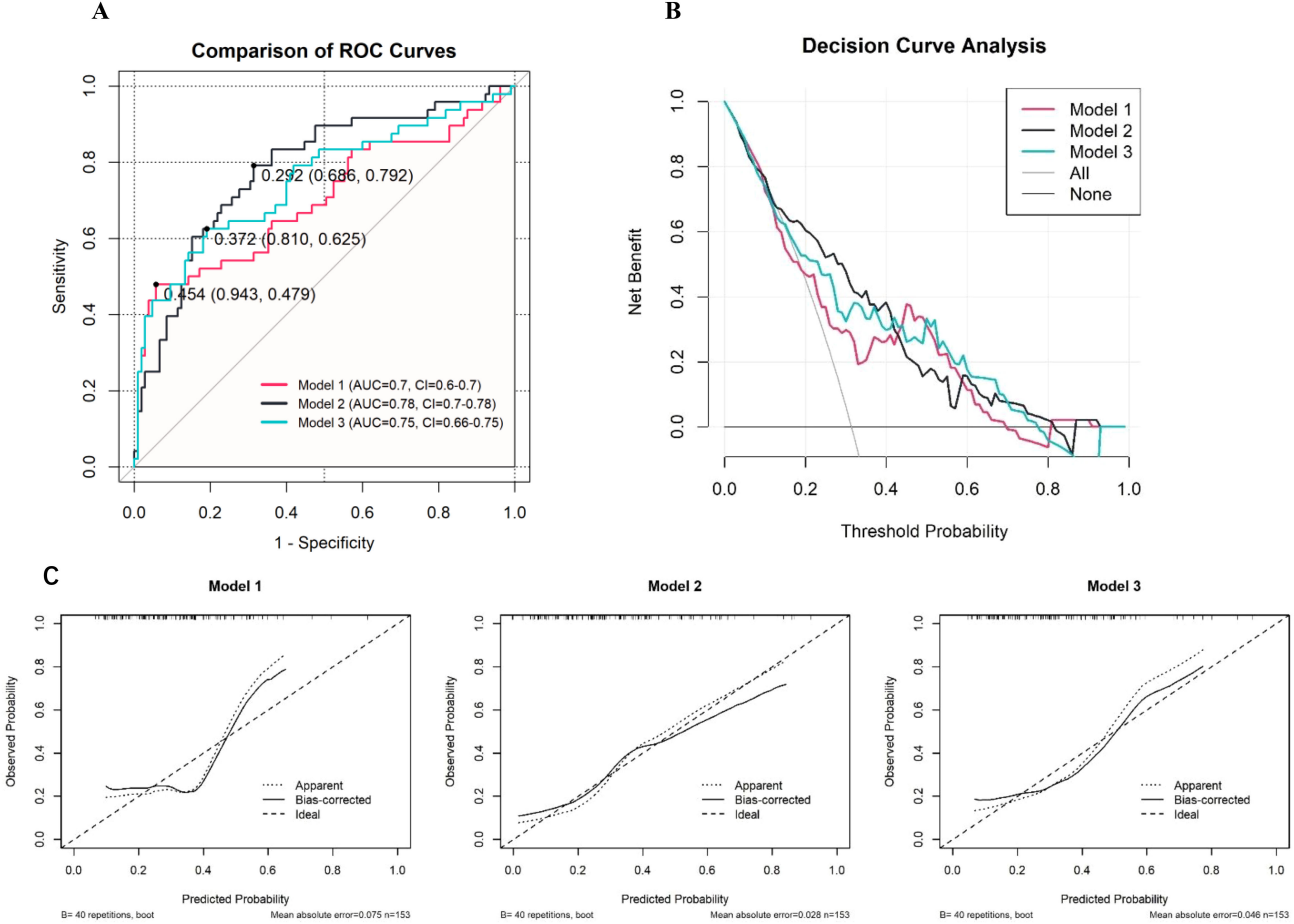
Performance Evaluation of Predictive Models for irAEs (**A**: ROC Curves, **B**: Decision Curve Analysis, **C**: Calibration Curves).

In this study, owing to the relatively small sample size of only 153 cases, we opted for k-fold cross-validation instead of the holdout method for internal validation (12). This approach provides performance metrics for the three different models. Specifically, the first model had an ROC value of 0.627, with a sensitivity of 0.955 and a specificity of 0.300; the second model had an ROC value of 0.706, with a sensitivity of 0.877 and a specificity of 0.305; and the third model had an ROC value of 0.671, with a sensitivity of 0.867 and a specificity of 0.355 (**Figure 3A**). To further assess the performance of these models, we also used ROC curves, DCA (**Figure 3B**), and calibration curves (**Figure 3C**) (13). The second model achieved an area under the ROC curve (AUC) of 0.78, indicating moderate predictive ability. While this level of discrimination suggests potential clinical utility, it is not ideal, and further refinement of the model is warranted. Future studies with larger external datasets may help optimize its predictive performance (14). T The inclusion criteria he DCA curves further demonstrated the net benefits of the three models across different thresholds, with the second model also showing greater clinical utility (15). Finally, calibration curves were used to evaluate the agreement between the predicted probabilities and actual outcomes, and all the models exhibited good calibration (16). Notably, the second model had the smallest deviation between the bias-corrected curve and the ideal curve. In conclusion, on the basis of the results of the k-fold cross-validation and the evaluation through various curves, the second model demonstrated the best performance in distinguishing and predicting the occurrence of irAEs, making it the most clinically valuable among the three models.

## Discussion

For patients with recurrent or metastatic NPC, traditional treatments, including radiotherapy and chemotherapy, often fail to achieve long-term disease control, leading researchers to explore alternative therapeutic strategies (17). In recent years, Immune Checkpoint Inhibitors (ICIs) particularly those targeting Programmed Death 1 (PD-1) and PD-L1, have emerged as promising options for the treatment of various cancers, including NPC (18). These inhibitors work by reversing the immunosuppressive effects in the tumor microenvironment, thus reactivating the immune system’s T cells to combat tumor growth (19). While some patients have demonstrated substantial clinical benefits from ICIs, the overall objective response rates (ORRs) remain modest, typically approximately 20–30% according to most studies (5). Moreover, the unpredictable and sometimes severe nature of irAEs presents additional challenges, highlighting the need for biomarkers to predict irAEs early in clinical practice (20).

Research has demonstrated that blood biomarkers, including the NLR and PLR, are significantly associated with the efficacy and adverse effects of ICIs (21). In non-small cell lung cancer, elevated NLR have been linked to poorer responses to immunotherapy and a greater risk of irAEs (4, 22). Similarly, studies have shown that in melanoma patients, higher NLR and PLR are associated with worse clinical outcomes and a greater incidence of irAEs (23). In gastric cancer, a low LMR has also been identified as a potential biomarker for predicting the occurrence of irAEs (24). Furthermore, in head and neck squamous cell carcinoma, higher serum Albumin (ALB) levels are correlated with a lower risk of irAEs, which may be related to the role of nutritional status in regulating the immune system (25). However, research on predicting irAEs in NPC remains limited.

This study included 153 patients with recurrent or metastatic NPC treated with PD-L1 inhibitors. Through systematic analysis, we explored factors associated with the occurrence of irAEs and developed a nomogram model to predict the occurrence. The results revealed that approximately 31.3% of patients experienced irAEs during treatment, with the highest incidence rates observed in endocrine-, digestive-, and cardiac-related adverse events, at 20.9%, 7.8%, and 6.5%, respectively. Specifically, the severity of most irAEs was classified as Grade 1 or 2, accounting for 87.5% (42/48) of all irAEs. However, 12.5% (6/48) of patients experienced severe irAEs of grade 3 or higher, particularly in the cardiac and digestive systems. These findings are consistent with the irAEs occurrence reported in other cancer, underscoring the need for special attention to specific system-related irAEs in NPC patients (26–28). This finding also highlights that irAEs in certain systems can progress to more severe degrees, aligning with current reports on irAEs in cancer immunotherapy (29). In the univariate logistic regression analysis, we initially identified several variables that might be associated with the occurrence of irAEs and converted these numerical variables into binary categories for further analysis. The multivariate logistic regression analysis ultimately identified PD-L1 (OR = 0.432, 95% CI: 0.188–0.993, p = 0.048), FT4 (OR = 0.921, 95% CI: 0.851–0.996, p = 0.040), Na (OR = 2.461, 95% CI: 1.109–5.459, p = 0.027), and lymphocyte count (OR = 2.768, 95% CI: 1.052–7.285, p = 0.039) as independent predictors. These findings suggest that these biomarkers play crucial roles in the occurrence of irAEs and could serve as potential indicators for predicting irAEs in clinical practice (26, 30, 31). On the basis of these independent predictors, we constructed three different nomogram models via multivariate logistic regression, LASSO regression, and ridge regression techniques (32). Among these models, the second model, which selected the most relevant variables through a stepwise regression method, demonstrated the best predictive performance. Internal validation revealed that the second model achieved an area under the ROC curve (AUC) of 0.78, with a sensitivity of 0.877 and a specificity of 0.305, indicating good predictive ability. Additionally, DCA was used to evaluate the clinical net benefit of the model, with the results showing that the second model exhibited high clinical utility across different thresholds. Finally, calibration curve analysis validated the accuracy of the model, with the second model showing the smallest deviation between the predicted probabilities and observed outcomes, indicating high calibration and reliability. These findings provide important guidance for clinical management.

Compared with existing irAEs prediction models, most prior studies have focused on the analysis of a single biomarker, namely the NLR or the PLR. These studies typically assess the correlation between a single indicator and the occurrence of irAEs (33, 34). However, the limitation of this approach lies in the fact that a single biomarker often cannot fully capture the complex biological state of a patient, which may restrict its predictive power. In this study, we adopted a comprehensive approach, utilizing multivariate logistic regression, LASSO regression, and ridge regression to systematically identify multiple biomarkers closely associated with the occurrence of irAEs and constructed different multivariable prediction models. After comparing the predictive performance of the three models, our study highlights the unique roles of biomarkers, including PD-L1, FT4, Na, and lymphocyte count, which are less frequently considered together in the prediction of irAEs in NPC (35). By integrating these indicators, we can more accurately identify patients who are at greater risk of developing severe irAEs, thereby providing more effective guidance for clinical treatment. Additionally, our predictive models demonstrated good performance in internal validation, especially the second model, which achieved an area under the ROC curve (AUC) of 0.78—significantly greater than that of several previous models based on single biomarkers (11). In contrast, the AUCs of the prediction models in previous studies typically ranged from 0.6--0.7, indicating that their predictive capabilities may not be sufficient for widespread clinical application (22). By incorporating the LASSO and ridge regression techniques, our models effectively reduce the problem of overfitting, enhancing their stability and generalizability. These advantages make our models more clinically valuable in predicting the risk of irAEs in NPC patients undergoing immunotherapy. Moreover, our predictive models are not limited to predicting the occurrence of irAEs; they also explore the potential mechanisms behind these adverse events through multivariate analysis. For instance, a decrease in PD-L1 expression may be significantly associated with the occurrence of irAEs-a finding that is consistent with research in other cancer types but is reported for the first time in NPC patients (26, 29). Additionally, changes in FT4 and Na levels may play important roles in the development of irAEs, providing new insights for further investigations into the biological mechanisms of these adverse events (31, 36).

Our study demonstrates that the developed nomogram provides moderate predictive accuracy for irAEs in NPC patients. While the model’s performance metrics support its potential clinical use, its practical applicability must also be considered. The second model, which incorporates a broader range of clinical and laboratory parameters, requires more detailed input, potentially increasing time consumption. However, once key variables are obtained, the calculation process is relatively fast and can be performed using readily available statistical software or online tools. Future integration into electronic medical records (EMRs) could further enhance accessibility and ease of use in clinical settings. Additionally, automated computation within a user-friendly interface could minimize workload for clinicians, increasing the model’s real-world applicability.

Despite the significant progress made in predicting irAEs, this study has several limitations. First, A key limitation of our study is the relatively small sample size, with only 153 patients, which may limit the generalizability of the model. However, given the rarity of PD-L1 inhibitor-treated recurrent or metastatic NPC cases, obtaining a large sample size is challenging. Future research should involve larger sample sizes, preferably from multiple centers, to validate the model's applicability and reliability in a broader population. Second, this study focused primarily on NPC patients treated with PD-L1 inhibitors, so the model’s applicability to other ICIs or different cancer types remains to be validated. Additionally, the study employed k-fold cross-validation for internal validation, which performs well with small sample sizes but lacks external validation. Thus, future studies should conduct external validation in independent cohorts to ensure the broad clinical applicability of the model. Finally, the mechanisms underlying irAEs are complex and may be influenced by various factors. This study did not encompass all potential influencing factors, indicating a need for future research to integrate more biological and clinical data to further refine and optimize the predictive model.

## Conclusion

This study systematically analyzed 153 patients with recurrent metastatic NPC who were treated with PD-L1 inhibitors and successfully developed and validated three nomogram models for predicting the occurrence of irAEs. The results identified PD-L1, FT4, Na, and lymphocyte count as factors in predicting irAEs. Among the models, the second model demonstrated the best predictive performance, significantly enhancing both the accuracy and clinical utility of irAEs prediction. While the developed nomogram demonstrates promising predictive performance for irAEs in advanced NPC patients, further external validation is necessary to ensure its broader applicability. Future research should focus on validating the model in larger, multi-center cohorts and evaluating its potential utility across different malignancies.

## Data Availability

The raw data supporting the conclusions of this article will be made available by the authors, without undue reservation.

## Glossary

NPC: Nasopharyngeal Carcinoma
irAEs: Immune-related Adverse Events
PD-L1: Programmed Death-Ligand 1
ICIs: Immune Checkpoint Inhibitors
PFS: Progression-Free Survival
OS: Overall Survival
DCB: Durable Clinical Benefit
AEs: Adverse Events
ECOG: Eastern Cooperative Oncology Group
cHL: Classical Hodgkin Lymphoma
AHCT: Allogeneic Hematopoietic Cell Transplantation
ROC: Receiver Operating Characteristic
AUC: Area Under the Curve
DCA: Decision Curve Analysis
NLR: Neutrophil-to-Lymphocyte Ratio
PLR: Platelet-to-Lymphocyte Ratio
LMR: Lymphocyte-to-Monocyte Ratio
PAR: Platelet-to-Albumin Ratio
LASSO: Least Absolute Shrinkage and Selection Operator
Ridge: Ridge Regression
AIC: Akaike Information Criterion
KM: Kaplan-Meier
FT4: Free Thyroxine
Na: Sodium
K: Potassium
LDL: Low-Density Lipoprotein
TG: Triglycerides
PT: Prothrombin Time
APTT: Activated Partial Thromboplastin Time
EBV DNA: Epstein-Barr Virus DNA
PD: Progressive Disease
AP: Alkaline Phosphatase
CI: Confidence Interval
LR: Logistic Regression
RO: Radiation Oncology
TT: Thyroid Test
HGB: Hemoglobin
PLT: Platelet
WBC: White Blood Cell
RBC: Red Blood Cell
NK Cells: Natural Killer Cells
CD4/CD8: Cluster of Differentiation 4/8 Ratio

## References

[1] Chen YP Chan ATC Le QT Blanchard P Sun Y Ma J. Nasopharyngeal carcinoma. Lancet. (2019) 394:64–80. doi: 10.1016/s0140-6736(19)30956-0 31178151

[2] Wang FH Wei XL Feng J Li Q Xu N Hu XC. Efficacy, safety, and correlative biomarkers of toripalimab in previously treated recurrent or metastatic nasopharyngeal carcinoma: A phase II clinical trial (POLARIS-02). J Clin Oncol. (2021) 39:704–12. doi: 10.1200/jco.20.02712 PMC8078488 33492986

[3] Xu JY Wei XL Ren C Zhang Y Hu YF Li JY. Association of plasma Epstein-Barr virus DNA with outcomes for patients with recurrent or metastatic nasopharyngeal carcinoma receiving anti-programmed cell death 1 immunotherapy. JAMA Netw Open. (2022) 5:e220587. doi: 10.1001/jamanetworkopen.2022.0587 35230439 PMC8889459

[4] Wang H Yang R Liu D Li W. Association of pretreatment neutrophil-to-lymphocyte ratio with clinical outcomes in cancer immunotherapy: An evidence synthesis from 30 meta-analyses. Int Immunopharmacol. (2024) 132:111936. doi: 10.1016/j.intimp.2024.111936 38579566

[5] Bhardwaj PV Abdou YG. The evolving landscape of immune checkpoint inhibitors and antibody drug conjugates in the treatment of early-stage breast cancer. Oncologist. (2023) 28:832–44. doi: 10.1093/oncolo/oyad233 PMC11025387 37597245

[6] Shi Y Qin X Peng X Zeng A Li J Chen C. Efficacy and safety of KL-A167 in previously treated recurrent or metastatic nasopharyngeal carcinoma: a multicenter, single-arm, phase 2 study. Lancet Reg Health West Pac. (2023) 31:100617. doi: 10.1016/j.lanwpc.2022.100617 36879786 PMC9985015

[7] Jin S Liu H Yang J Zhou J Peng D Liu X. Development and validation of a nomogram model for cancer-specific survival of patients with poorly differentiated thyroid carcinoma: A SEER database analysis. Front Endocrinol (Lausanne). (2022) 13:882279. doi: 10.3389/fendo.2022.882279 36176465 PMC9513392

[8] Lu J Liao J Chen Y Li J Huang X Zhang H. Risk factor analysis and prediction model for papillary thyroid carcinoma with lymph node metastasis. Front Endocrinol (Lausanne). (2023) 14:1287593. doi: 10.3389/fendo.2023.1287593 38027220 PMC10646784

[9] Basu A Ghosh A Jaenada M Pardo L. Robust adaptive LASSO in high-dimensional logistic regression. In: Statistical Methods & Applications Springer, Berlin, Germany. (2024). doi: 10.1007/s10260-024-00760-2

[10] Belhechmi S Bin R Rotolo F Michiels S. Accounting for grouped predictor variables or pathways in high-dimensional penalized Cox regression models. BMC Bioinf. (2020) 21:277. doi: 10.1186/s12859-020-03618-y PMC7331150 32615919

[11] Li X Tong L Wang S Yu J Lu B Wang Q. Integration of clinical and blood parameters in risk prognostication for patients receiving immunochemotherapy for extensive stage small cell lung cancer: real-world data from two centers. BMC Med. (2024) 22:381. doi: 10.1186/s12916-024-03612-8 39256789 PMC11389556

[12] White J Power SD. k-fold cross-validation can significantly over-estimate true classification accuracy in common EEG-based passive BCI experimental designs: an empirical investigation. Sensors. (2023) 23:6077. doi: 10.3390/s23136077 37447926 PMC10346713

[13] Yuan C Zou S Wang K Hu Z. Establishment and external validation of prognosis prediction nomogram for patients with distant metastatic intrahepatic cholangiocarcinoma: based on a large population. BMC Cancer. (2024) 24:227. doi: 10.1186/s12885-024-11976-6 38365630 PMC10874087

[14] Hayes T Baraldi AN Coxe S. Random forest analysis and lasso regression outperform traditional methods in identifying missing data auxiliary variables when the MAR mechanism is nonlinear (p.s. Stop using Little’s MCAR test). Behav Res Methods. (2024) 56(8):8608–39. doi: 10.3758/s13428-024-02494-1 39251529

[15] Piovani D Sokou R Tsantes AG Vitello AS Bonovas S. Optimizing clinical decision making with decision curve analysis: insights for clinical investigators. Healthcare. (2023) 11:2244. doi: 10.3390/healthcare11162244 37628442 PMC10454914

[16] Van Calster B McLernon DJ van Smeden M Wynants L Steyerberg EW Bossuyt P. Calibration: the Achilles heel of predictive analytics. BMC Med. (2019) 17:230. doi: 10.1186/s12916-019-1466-7 31842878 PMC6912996

[17] Zhu Y Liu K Ding D Wang K Liu X Tan X. Chemo-immunotherapy regimes for recurrent or metastatic nasopharyngeal carcinoma: A network meta-analysis and cost-effectiveness analysis. Front Pharmacol. (2022) 13:858207. doi: 10.3389/fphar.2022.858207 35668931 PMC9163401

[18] Ma BBY Lim WT Goh BC Hui EP Lo KW Pettinger A. Antitumor activity of nivolumab in recurrent and metastatic nasopharyngeal carcinoma: an international, multicenter study of the mayo clinic phase 2 consortium (NCI-9742). J Clin Oncol. (2018) 36:1412–8. doi: 10.1200/jco.2017.77.0388 PMC5941615 29584545

[19] Pang L Xie M Ma X Huang A Song J Yao J. Clinical characteristics and therapeutic effects of checkpoint inhibitor-related pneumonitis in patients with non-small cell lung cancer. BMC Cancer. (2023) 23:203. doi: 10.1186/s12885-023-10649-0 36869304 PMC9983156

[20] Ponvilawan B Khan AW Subramanian J Bansal D. Non-invasive predictive biomarkers for immune-related adverse events due to immune checkpoint inhibitors. Cancers. (2024) 16:1225. doi: 10.3390/cancers16061225 38539558 PMC10968874

[21] Chen C Yang H Cai D Xiang L Fang W Wang R. Preoperative peripheral blood neutrophil-to-lymphocyte ratios (NLR) and platelet-to-lymphocyte ratio (PLR) related nomograms predict the survival of patients with limited-stage small-cell lung cancer. Transl Lung Cancer Res. (2021) 10:866–77. doi: 10.21037/tlcr-20-997 PMC7947425 33718028

[22] Diem S Schmid S Krapf M Flatz L Born D Jochum W. Neutrophil-to-Lymphocyte ratio (NLR) and Platelet-to-Lymphocyte ratio (PLR) as prognostic markers in patients with non-small cell lung cancer (NSCLC) treated with nivolumab. Lung Cancer. (2017) 111:176–81. doi: 10.1016/j.lungcan.2017.07.024 28838390

[23] Tan S Zheng Q Zhang W Zhou M Xia C Feng W. Prognostic value of inflammatory markers NLR, PLR, and LMR in gastric cancer patients treated with immune checkpoint inhibitors: a meta-analysis and systematic review. Front Immunol. (2024) 15:1408700. doi: 10.3389/fimmu.2024.1408700 39050856 PMC11266030

[24] Ruan DY Chen YX Wei XL Wang YN Wang ZX Wu HX. Elevated peripheral blood neutrophil-to-lymphocyte ratio is associated with an immunosuppressive tumour microenvironment and decreased benefit of PD-1 antibody in advanced gastric cancer. Gastroenterol Rep (Oxf). (2021) 9:560–70. doi: 10.1093/gastro/goab032 PMC8677531 34925853

[25] Suzuki S Taguchi Y Kitabayashi T Sato N Kaya H Abe T. Serum albumin as an independent predictor of long-term survival in patients with recurrent and metastatic head and neck squamous cell carcinoma treated with nivolumab. J Clin Med. (2024) 13:2456. doi: 10.3390/jcm13092456 38730986 PMC11084251

[26] Ferris RL Blumenschein G Jr. Fayette J Guigay J Colevas AD Licitra L. Nivolumab for recurrent squamous-cell carcinoma of the head and neck. N Engl J Med. (2016) 375:1856–67. doi: 10.1056/NEJMoa1602252 PMC5564292 27718784

[27] Mehra R Seiwert TY Gupta S Weiss J Gluck I Eder JP. Efficacy and safety of pembrolizumab in recurrent/metastatic head and neck squamous cell carcinoma: pooled analyses after long-term follow-up in KEYNOTE-012. Br J Cancer. (2018) 119:153–9. doi: 10.1038/s41416-018-0131-9 PMC6048158 29955135

[28] Cramer JD Burtness B Ferris RL. Immunotherapy for head and neck cancer: Recent advances and future directions. Oral Oncol. (2019) 99:104460. doi: 10.1016/j.oraloncology.2019.104460 31683169 PMC7749717

[29] Postow MA Sidlow R Hellmann MD. Immune-related adverse events associated with immune checkpoint blockade. N Engl J Med. (2018) 378:158–68. doi: 10.1056/NEJMra1703481 29320654

[30] Shankar B Zhang J Naqash AR Forde PM Feliciano JL Marrone KA. Multisystem immune-related adverse events associated with immune checkpoint inhibitors for treatment of non–small cell lung cancer. JAMA Oncol. (2020) 6:1952–6. doi: 10.1001/jamaoncol.2020.5012 PMC7596677 33119034

[31] Hsu C Lee SH Ejadi S Even C Cohen RB Le Tourneau C. Safety and antitumor activity of pembrolizumab in patients with programmed death-ligand 1-positive nasopharyngeal carcinoma: results of the KEYNOTE-028 study. J Clin Oncol. (2017) 35:4050–6. doi: 10.1200/jco.2017.73.3675 28837405

[32] Wang Q Qiao W Zhang H Liu B Li J Zang C. Nomogram established on account of Lasso-Cox regression for predicting recurrence in patients with early-stage hepatocellular carcinoma. Front Immunol. (2022) 13:1019638. doi: 10.3389/fimmu.2022.1019638 36505501 PMC9726717

[33] Takada S Murooka H Tahatsu K Yanase M Umehara K Hashishita H. Identifying early predictive markers for immune-related adverse events in nivolumab-treated patients with renal cell carcinoma and gastric cancer. Asian Pac J Cancer Prev. (2022) 23:695–701. doi: 10.31557/APJCP.2022.23.2.695 35225483 PMC9272606

[34] Lu H-R Zhu P-F Deng Y-Y Chen Z-L Yang L. Predictive value of NLR and PLR for immune-related adverse events: a systematic review and meta-analysis. Clin Trans Oncol. (2024) 26:1106–16. doi: 10.1007/s12094-023-03313-3 37682501

[35] Xu R Wong CHL Chan KSK Chiang CL. PD-L1 expression as a potential predictor of immune checkpoint inhibitor efficacy and survival in patients with recurrent or metastatic nasopharyngeal cancer: a systematic review and meta-analysis of prospective trials. Front Oncol. (2024) 14:1386381. doi: 10.3389/fonc.2024.1386381 38887234 PMC11180873

[36] Xu J-Y Wei X-L Ren C Zhang Y Hu Y-F Li J-Y. Association of plasma Epstein-Barr virus DNA with outcomes for patients with recurrent or metastatic nasopharyngeal carcinoma receiving anti–programmed cell death 1 immunotherapy. JAMA Network Open. (2022) 5:e220587–e. doi: 10.1001/jamanetworkopen.2022.0587 PMC8889459 35230439

