# Corrigendum: Development and validation of nomogram models for predicting immune-related adverse events in recurrent and metastatic nasopharyngeal carcinoma patients treated with PD-L1 inhibitors

**DOI:** 10.3389/fonc.2025.1610079

**Published:** 2025-05-15

**Authors:** Mengyuan Liu, Zheran Liu, Shuangshuang He, Yiyan Pei, Shihong Xu, Junyou Ge, Yan Qing, Youneng Wei, Ye Chen, Ping Ai, Xingchen Peng

**Affiliations:** ^1^ Division of Head & Neck Tumor Multimodality Treatment, Cancer Center, West China Hospital, Sichuan University, Chengdu, China; ^2^ Department of Targeting Therapy & Immunology, Cancer Center, West China Hospital, Sichuan University, Chengdu, China; ^3^ Sichuan Kelun-Biotech Biopharmaceutical Co. Ltd., Chengdu, China; ^4^ Division of Abdominal Tumor Multimodality Treatment, Department of Radiation Oncology, Cancer Center, West China Hospital, Sichuan University, Chengdu, China

**Keywords:** NPC, irAEs, PD-L1 inhibitors, biomarkers, nomogram

In the published article “Shi Y, Qin X, Peng X, Zeng A, Li J, Chen C, et al. Efficacy and safety of KL-A167 in previously treated recurrent or metastatic nasopharyngeal carcinoma: a multicenter, single-arm, phase 2 study. Lancet Reg Health West Pac, 2023, 31: 100617” was not cited in the article. The citation has now been inserted in **Methods**, *Data source*, Paragraph 1 and should read:

“This study used data from an open-label, multicenter phase 2 clinical trial conducted between 2017 and 2019 at 42 hospitals in China involving 153 patients with NPC (**Supplementary Figure 1**) (6)”.

In the published article, there was an error in **Table 1** as published. In the ECOG performance status row, the current version displays the numerical coding used for statistical modeling (i.e., 1 and 2). This should be revised to reflect the actual clinical values (ECOG = 0 and ECOG = 1), as presented to clinicians. The corrected **Table 1** and its caption “Baseline characteristics of patients with and without immune-related adverse events (irAEs)” appear below.

**Table 1:** Baseline characteristics of patients with and without immune-related adverse events (irAEs).

Variable	Overall (N=153)	Without irAEs (N=105)	With irAEs (N=48)	p value
Age (Mean [SD])	47.56 (9.81)	48.03 (10.12)	46.54 (9.12)	0.386
Body Mass Index (kg/m^2^)				
<18.5	29 (19.0%)	17 (16.2%)	12 (25.0%)	0.260
18.5–23.9	92 (60.1%)	63 (60.0%)	29 (60.4%)	
>23.9	32 (20.9%)	25 (23.8%)	7 (14.6%)	
ECOG_PS				
0	59 (38.6%)	42 (40.0%)	17 (35.4%)	0.718
1	94 (61.4%)	63 (60.0%)	31 (64.6%)	
Gender				
Male	125 (81.7%)	86 (81.9%)	39 (81.2%)	1.000
Female	28 (18.3%)	19 (18.1%)	9 (18.8%)	
Tumor Stage				
T0–T2	52 (34.0%)	42 (40.0%)	10 (20.8%)	0.059
T3–T4	50 (32.7%)	30 (28.6%)	20 (41.7%)	
Tx	51 (33.3%)	33 (31.4%)	18 (37.5%)	
Node Stage				
N0–N2	84 (54.9%)	55 (52.4%)	29 (60.4%)	0.537
N3	26 (17.0%)	20 (19.0%)	6 (12.5%)	
Nx	43 (28.1%)	30 (28.6%)	13 (27.1%)	
Liver Metastasis				
No	82 (53.6%)	54 (51.4%)	28 (58.3%)	0.535
Yes	71 (46.4%)	51 (48.6%)	20 (41.7%)	

In the published article, there was an error in **Table 2** as published. A portion of the fatigue-related data was inadvertently omitted during table compilation. Upon verification, the total number of fatigue events, including Grade 1–2, should be 4. The corrected **Table 2** and its caption “Incidence and severity of immune-related adverse events (irAEs) among patients” appear below.

**Table 2:** Incidence and severity of immune-related adverse events (irAEs) among patients.

irAEs	Summary	Grade1_2	Grade3_4
Any irAEs	48 (31.3%)	42 (27.4%)	6 (3.9%)
Endocine irAEs	32 (20.9%)	32 (20.9%)	0
Cardiac irAEs	10 (6.5%)	8 (5.2%)	2 (1.3%)
Digestive System irAEs	12 (7.8%)	9 (5.9%)	3 (1.9%)
Hematology irAEs	6 (3.9%)	4 (2.6%)	2 (1.3%)
Dermatological irAEs	5 (3.2%)	5 (3.2%)	0
Fatigue	4 (2.6%)	4 (2.6%)	0
Renal irAEs	2 (1.3%)	2 (1.3%)	0
Metabolism irAEs	6 (3.9%)	5 (3.2%)	1 (0.7%)

In the published article, there was an error in Figure 4 as published. After publication, we realized that the survival curve presented in Figure 4 overlaps with data previously published by Dr. Shihong Xu in *Oral Oncology* (2025; https://doi.org/10.1016/j.oraloncology.2024.107161). Dr. Xu is one of our research collaborators. Although using the same database, we analysed different markers. We focused on the predictive modeling. We acknowledge that including this figure may result in unintended duplication. To maintain clarity and proper attribution, we respectfully request the removal of Figure 4 from the published article.

In the published article, there was an error in Supplementary Figure 2. Upon verification, the total number of fatigue events, including Grade 1–2, should be 4.

In the published article, there was an error. As in the case of Figure 4, we removed the sentence discussing survival analysis, which overlapped with results already reported by Dr. Shihong Xu in *Oral Oncology* (2025). Therefore, we do not elaborate on this point further in the current article.

A correction has been made to **Discussion**, Paragraph 3. This sentence previously stated:

“Further KM survival curve analysis revealed that patients who experienced irAEs had better PFS, suggesting that the occurrence of irAEs may be associated with a better treatment response (**Figure 4**).”

This sentence has been removed.

The authors apologize for this error and state that this does not change the scientific conclusions of the article in any way. The original article has been updated.

