# Supplementary material for: Development and validation of nomogram models for predicting immune-related adverse events in recurrent and metastatic nasopharyngeal carcinoma patients treated with PD-L1 inhibitors
Source: Front Oncol. 2025 Mar 13;15:1539514. doi: 10.3389/fonc.2025.1539514 (PMC11966434; doi:10.3389/fonc.2025.1539514)
Supplement: Supplementary file 1 [file SupplementaryFile1.docx]

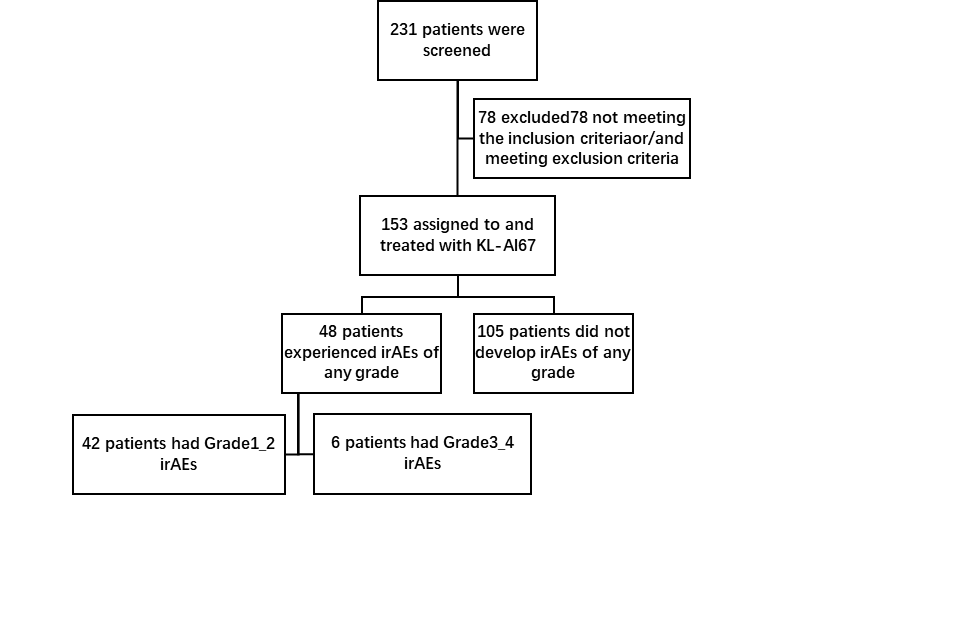


Supplementary Figure 1 Flowchart of patient screening, enrollment, and incidence of irAEs


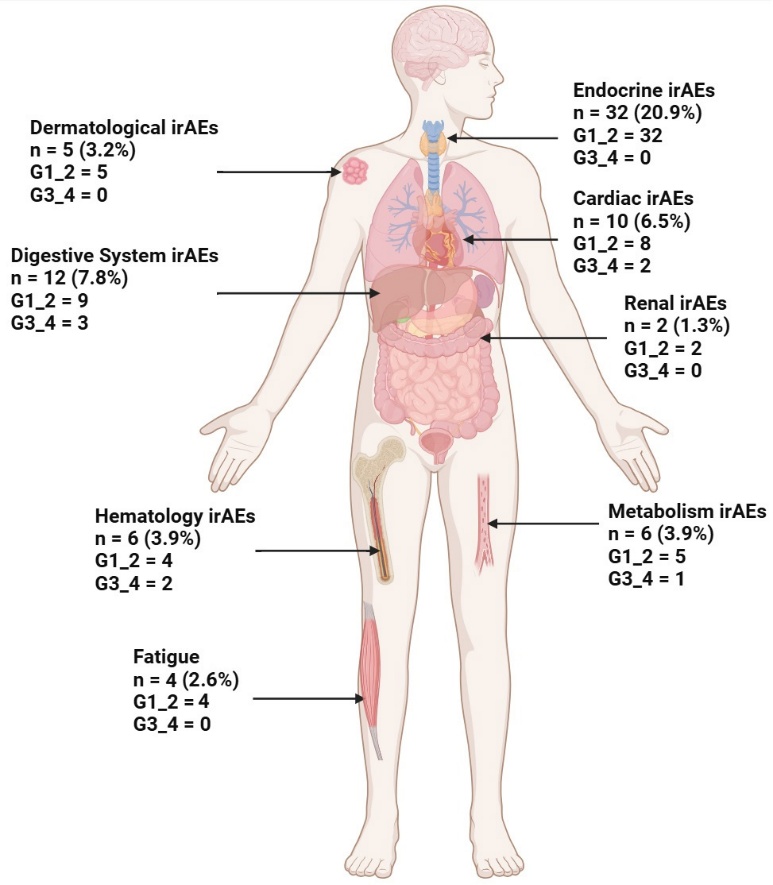


Supplementary Figure 2 Distribution and severity of immune-related adverse events (irAEs) across different organ systems
